# COVID-19: A scholarly production dataset report for research analysis

**DOI:** 10.1016/j.dib.2020.106178

**Published:** 2020-08-19

**Authors:** Breno Santana Santos, Ivanovitch Silva, Marcel da Câmara Ribeiro-Dantas, Gisliany Alves, Patricia Takako Endo, Luciana Lima

**Affiliations:** a Universidade Federal do Rio Grande do Norte (UFRN), Rio Grande do Norte, Brazil; b Núcleo de Pesquisa e Prática em Inteligência Competitiva (NUPIC), Universidade Federal de Sergipe (UFS), Itabaiana, SE, Brazil; c Institut Curie (UMR168), Sorbonne Université (EDITE), Paris, France; d Universidade de Pernambuco (UPE), Pernambuco, Brazil

**Keywords:** COVID-19, SARS-CoV-2, Pandemic, Data Science, Bibliometrics, Scientometrics

## Abstract

COVID-2019 has been recognized as a global threat, and several studies are being conducted in order to contribute to the fight and prevention of this pandemic. This work presents a scholarly production dataset focused on COVID-19, providing an overview of scientific research activities, making it possible to identify countries, scientists and research groups most active in this task force to combat the coronavirus disease. The dataset is composed of 40,212 records of articles’ metadata collected from Scopus, PubMed, arXiv and bioRxiv databases from January 2019 to July 2020. Those data were extracted by using the techniques of Python Web Scraping and preprocessed with Pandas Data Wrangling. In addition, the pipeline to preprocess and generate the dataset are versioned with the Data Version Control tool (DVC) and are thus easily reproducible and auditable.

## Specifications Table

**Table:** 

Subject	Infectious Diseases.
Specific subject area	Bibliometrics, Scientometrics, Complex Network Analysis, Data Science.
Type of data	Text file (CSV format).
How data were acquired	The Jupyter Notebooks and Python scripts to collect and process the data is available at https://github.com/breno-madruga/dib-covid-dataset/.
	Python Web Scraping tools (Scrapy) [1]
	Pymed library to collect PubMed data [2]
	Pybliometrics library to collect Scopus data [3]
	bioRxiv URL (https://connect.biorxiv.org/relate/content/181)
	arXiv URL (https://arxiv.org/covid19search)
Data format	Raw. The data were retrieved from arXiv [4], bioRxiv/medRxiv [5], PubMed [6], and Scopus [7] databases, processed and made available in a text file (CSV format).
Parameters for data collection	The searching parameters and data collection used in the following databases:
	• arXiv: https://arxiv.org/covid19search
	• bioRxiv/medRxiv: https://connect.biorxiv.org/relate/content/181
	• PubMed: (covid-19 OR coronavirus disease 2019 OR 2019-ncov OR novel coronavirus OR sars-cov-2 OR novel coronavirus pneumonia OR coronavirus) AND (2019[Date - Publication]:2020[Date - Publication]) AND (english[Language])
	• Scopus: TITLE-ABS-KEY(“covid-19” OR “coronavirus disease 2019” OR “2019-ncov” OR “novel coronavirus” OR “sars-cov-2” OR “novel coronavirus pneumonia” OR “coronavirus”) AND PUBYEAR > 2018 AND LANGUAGE(english)
Description of data collection	The metadata of articles related to COVID-2019 from January 2019 to July 2020 were collected from arXiv, bioRxiv/medRxiv, PubMed and Scopus databases, and the tools and techniques of Python Web Scraping [1] and Pandas Data Wrangling [8] were used to build a scholarly production dataset.
Data source location	Online at https://data.mendeley.com/datasets/kx7wwc8dzp/.
Data accessibility	The dataset is hosted at https://data.mendeley.com/datasets/kx7wwc8dzp/.

## Value of Data

• This dataset can be used by other researchers to implement automatic mechanisms (through Natural Language Processing, for instance) to extract insights contained on the metadata (e.g., abstracts and keywords) of scholarly studies;
• This dataset can also be used together with other datasets (e.g. Publon and Google Scholar) in order to get a more accurate overview of research related to COVID-19 and identify possible research gaps that have not yet been explored to combat COVID-19;
• Several insights can be extracted from the relationships among various entities (e.g. drugs, researchers and their affiliations) applying techniques of Complex Network Analysis in this dataset;
• The most influential researchers or research groups can be identified to initiate new possible collaborations or task forces to combat COVID-19 pandemic.

## 1 Data description

The dataset available in this paper is composed of 40,212 records of metadata about the publications related to COVID-19. Such data were collected from Scopus [7], PubMed [6], arXiv [4] and bioXiv/medRxiv [5] databases, and correspond to productions whose publication year is 2019 or 2020, and were published, indexed or made available until 07/02/2020 (date of data collection).

Tables 1, 2, 3 and 4 present the features contained in these specific datasets. It is worth mentioning that the final dataset practically has the same features as the Scopus dataset, except for having the “data_source” feature, which characterizes the original dataset (arXiv, bioRxiv, medRxiv, PubMed or Scopus) of a record.

**Table 1:** The metadata of articles contained in the PubMed dataset.

**Feature**	**Description**
pubmed_id	The MEDLINE identifier of a manuscript.
doi	The DOI of a manuscript.
title	The title of a manuscript.
abstract	The abstract of a manuscript.
publication_date	The date of publication of a manuscript.
author_affil	A list of Python dictionaries that contains the authors and their affiliations’ information in a manuscript.
auth_keywords	A list of authors-provided keywords contained in a manuscript.
vehicle_name	The name of source where a manuscript was published.

**Table 2:** The features of articles contained in the Scopus dataset.

**Feature**	**Description**
id	The identifier key of a manuscript.
doi	The DOI of a manuscript.
pubmed_id	The MEDLINE identifier of a manuscript.
title	The title of a manuscript.
abstract	The abstract of a manuscript.
publication_date	The date of publication of a manuscript.
citation_num	The number of citation of a manuscript.
language	The language of a manuscript.
production_type	The type of source where a manuscript was published.
source_type	The type of source where a manuscript was published (short version of “production_type”).
auth_keywords	A list of authors-provided keywords contained in a manuscript.
index_terms	A list of index terms (these are just one category of those Scopus provides in the web version).
subject_areas	The research fields or subject areas related to a manuscript.
authors	A list of Python dictionaries that contains the authors/researchers’ information (Scopus ID and complete name) of a manuscript.
affiliations	A list of Python dictionaries that contains the authors affiliations’ information (Scopus ID, name and country) of a manuscript.
author_affil	A list of Python dictionaries that contains the authors and their affiliations’ information (authors’ Scopus ID, authors’ complete name, affiliations’ Scopus ID, affiliations’ names and affiliations’ countries) in a manuscript. In summary, it is a combination of the “authors” and “affiliations” features.
vehicle_name	The name of source where a manuscript was published.
publisher	The publisher’s name of a manuscript.
issn	The ISSN belonging to the “vehicle_name”. If E-ISSN is known to Scopus, this returns both ISSN and E-ISSN in random order separated by blank space.
ref_count	The number of references of a manuscript.
references	A list of Python dictionaries that contains the references’ information (Scopus ID, title, DOI and authors) of a manuscript.

**Table 3:** The metadata of articles contained in the arXiv dataset.

**Feature**	**Description**
id	The identifier key of a manuscript.
subject_areas	The research fields or subject areas related to a manuscript.
title	The title of a manuscript.
authors	A list with the authors/researchers’ complete name of a manuscript.
abstract	The abstract of a manuscript.
publication_date	The date of the last update of a manuscript.

**Table 4:** The metadata of articles contained in the bioRxiv/medRxiv dataset.

**Feature**	**Description**
id	The identifier key of a manuscript.
doi	The DOI of a manuscript.
title	The title of a manuscript.
abstract	The abstract of a manuscript.
publication_date	The date of publication of a manuscript.
author_affil	A list of Python dictionaries that contains the authors and their affiliations’ information in a manuscript.
source	This feature indicates whether a manuscript was extracted from bioRxiv or medRxiv platforms.

## 2 Experimental design, materials and methods

**Fig. 1:**
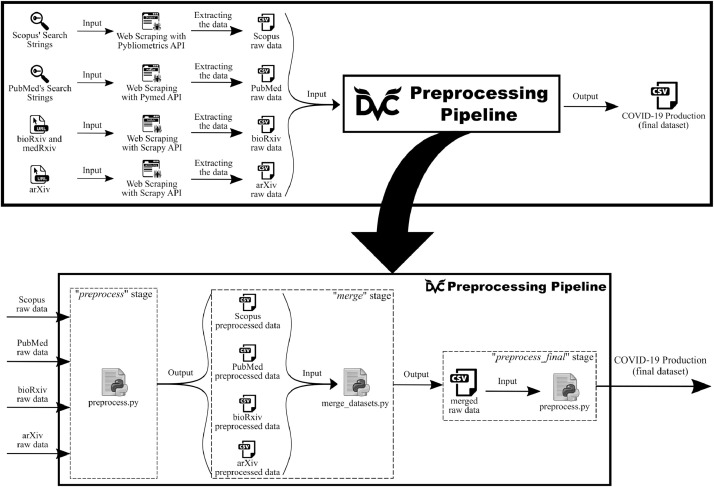
Methodology of COVID-19 Production Data Collecting.

The process of data collection can be seen in Fig. 1. For each database, a specific dataset was generated using Web Scraping tools and techniques [1] to collect its respective metadata. Next, using Pandas tool [8], all specific datasets were preprocessed (data cleaning and normalization, e.g.) and merged, as well as the duplicated records were removed, thus, generating our new dataset.

A pipeline was created with DVC, the Data Version Control tool^1^, to preprocess and merge these specific datasets, and it also generates our final dataset after the last stage (see Fig. 1). DVC provides data science workflow reproducibility and consistency, and it is Git-compatible, offering lock-free, local branching, and versioning. Furthermore, DVC is used to version data and data pipelines, following the same rationale used to version source code [9]. The scripts for the preprocessing and merging stages were written in the Python programming language^2^.

### 2.1 PubMed and Scopus databases

For PubMed [6] and Scopus [7] databases, the set of keywords were defined from Lou et al. [10] and by the search string “covid” used on the DeSC platform [11]. The most characteristic and significant keywords were chosen by two health professionals, which were used to collect the data in these databases. The search strings used were:

• **PubMed:** (covid-19 OR coronavirus disease 2019 OR 2019-ncov OR novel coronavirus OR sars-cov-2 OR novel coronavirus pneumonia OR coronavirus) AND (2019[Date - Publication]:2020[Date - Publication]) AND (english[Language]);
• **Scopus:** TITLE-ABS-KEY(“covid-19” OR “coronavirus disease 2019” OR “2019-ncov” OR “novel coronavirus” OR “sars-cov-2” OR “novel coronavirus pneumonia” OR “coronavirus”) AND PUBYEAR > 2018 AND LANGUAGE(english).

We also applied filters of year and language, i.e., the selected articles were published in the years 2019 and 2020, as well as they should be in English. After the definition of Scopus’ and PubMed’s search strings, the data of these databases were collected from the *Pybliometrics* [3] (Scopus) and *Pymed* [2] (PubMed) libraries. For the step of data wrangling, the Pandas [8] library was used to preprocess these data and generate the Scopus’ and PubMed’s datasets.

### 2.2 arXiv and bioRxiv databases

The process of collecting scholarly articles related to COVID-19, which were registered on the arXiv [4] and bioRxiv [5] platforms was performed as follows: in the homepage of the arXiv platform, there are two hyperlinks that point to the listing of these articles (https://arxiv.org/covid19search and https://connect.biorxiv.org/relate/content/181), and from these hyperlinks, the tools and techniques of Python Web Scraping [1], especially the Scrapy library^3^, were used to collect the metadata of these scholarly studies. Again, the Pandas library was used to preprocess these metadata and generate the arXiv’s and bioRxiv’s datasets. It is worth mentioning that the bioRxiv dataset has several articles belonging to both bioRxiv and medRxiv platforms.

Using the Pandas library, all aforementioned datasets were merged, and the duplicated records were removed. As the Scopus dataset is the richest in features/columns, it served as the basis for the creation of the final dataset. For records from arXiv’s and bioRxiv’s datasets, the duplicated articles were identified by the “title” feature, while, for the PubMed dataset, the duplicates were identified by the “title” and “pubmed_id” features. Moreover, a extra preprocessing step was perform at the final dataset after the merging processing, aiming to warranty the data consistency and integrity.

**Fig. 2:**
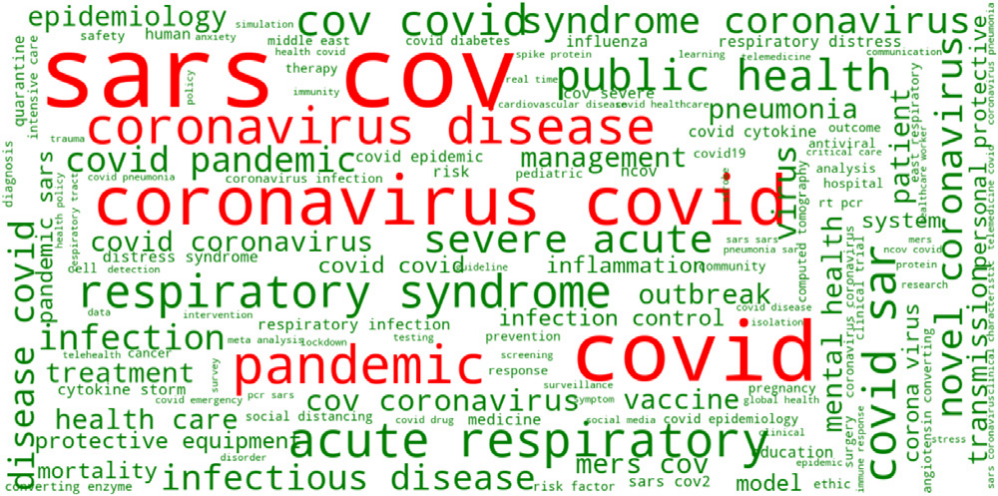
Main topics of COVID-19 Production Data.

As stated earlier, it has enormous potential to extract knowledge and several insights to support in combating the pandemic, for example, in Figure 2, the main topics of research related to COVID-19 can be extracted using Natural Language Processing techniques.

It is important to highlight that researchers that are not familiar with the Python ecosystem for Data Science and version control technologies (Git, GitHub and DVC) can directly access the data in CSV format available at Data Mendeley (https://data.mendeley.com/datasets/kx7wwc8dzp/). In addition, for those who are familiar and interested in more details about the acquisition and the preprocessing of the dataset, the pipeline and source codes (Jupyter Notebooks and Python scripts) are available at GitHub (https://github.com/breno-madruga/dib-covid-dataset/) and mirrored at DAGsHub^4^, having at their disposal the potential and advantages of the Python ecosystem for Data Science and the Git and DVC technologies.

## Declaration of Competing Interest

The authors declare that they have no known competing financial interests or personal relationships that have, or could be perceived to have, influenced the work reported in this article.

## Appendix A Supplementary materials

Supplementary Data S1 Supplementary Raw Research Data. This is open data under the CC BY license http://creativecommons.org/licenses/by/4.0/ Supplementary Data S1

## Footnotes

1 https://dvc.org/

2 https://www.python.org

3 https://scrapy.org

4 https://dagshub.com/breno-madruga/dib-covid-dataset/
